# BioMedStatX – Statistical workflows for reliable biomedical data analysis

**DOI:** 10.1016/j.mex.2025.103776

**Published:** 2025-12-17

**Authors:** Philipp Krumm, Nicole Böttcher, Richard Ottermanns, Thomas Pufe, Athanassios Fragoulis

**Affiliations:** a Department of Anatomy and Cell Biology, Uniklinik RWTH Aachen, 52074 Aachen, Germany; b Institute for Environmental Research, RWTH Aachen University, 52074 Aachen, Germany

**Keywords:** Statistics, Python, Statistical analysis, Statistical software, Open science, Biomedical research tools, User-friendly tools, Good scientific practice

## Abstract

Robust statistical analysis is essential for scientific validity and to ensure good scientific practice. Yet many researchers, especially in biomedical fields, struggle with checking assumptions, selecting the correct tests, and interpreting results. These obstacles can lead to misleading conclusions and undermine scientific progress.

BioMedStatX explicitly addresses these issues by ensuring that the implemented workflows exclude the use of inadequate statistical tests. This Python-based desktop application features an intuitive graphical interface that automatically selects appropriate statistical tests based on the data and its characteristics, ensuring that users, even with minor statistical training, follow a statistically valid workflow.

Users can import Excel or CSV files, select groups and let BioMedStatX manage the rest: from outlier detection, assumption checks and guided data transformations to test execution (parametric or non-parametric) and guided post-hoc analyses. Results are exported in a structured Excel workbook including a decision tree that visualizes each analytical step, and customizable plots are exported as SVG-/PNG-files.

By embedding statistical expertise directly into the software, BioMedStatX prevents invalid analysis paths, increases transparency, and enables reproducibility.

## Graphical abstract

Graphical abstract was uploaded as separate file.

**Figure:**
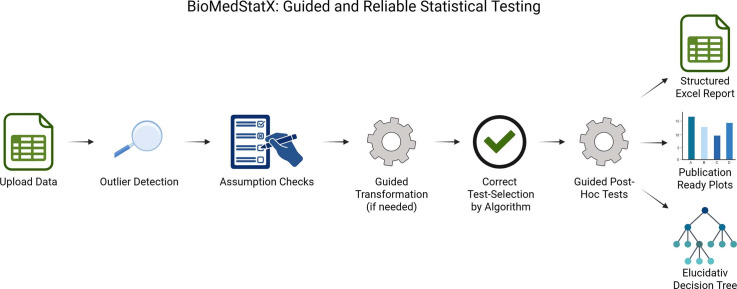

## Specifications table

**Table:** 

**Subject area**	Mathematics and Statistics
**More specific subject area**	Statistical Software
**Name of your method**	BioMedStatX
**Name and reference of original method**	not applicable
**Resource availability**	Software and Backend: https://github.com/philippkrumm/BioMedStatX.git The GitHub repository provides contribution guidelines, structured issue tracking, and documentation to support transparent open-source development. Users can report bugs or feature requests through the Issues tab, and external contributions follow a standard fork-and-pull-request workflow to ensure reproducibility and code quality.

## Background

Biomedical research leads to various data from experiments that were carried out in the laboratory. Proving that the results of experiments are not observed by chance is important to contribute to science. In statistics, the significance level is the value that indicates how likely it is that an observed difference or correlation between one and another sample is not random but also applies to the population [1]. Statistical analysis in biomedical research has two main branches, parametric tests and non-parametric tests. Choosing the correct branch is a critical step and is based on the prerequisites of the respective data. Parametric tests, e.g. *t*-test or ANOVA, can only be performed if the data are normally distributed and fulfill the condition of homoscedasticity.

If these assumptions fail, the data can be transformed and reanalyzed. If this also fails, a non-parametric test, such as the Mann-Whitney-U or Kruskal-Wallis test, can be applied [2]. The selection of an inappropriate test for the analysis of a dataset can lead to the misinterpretation of results, based on the significances that resulted from the analysis [3]. Multiple studies revealed various types of statistical errors made in publications. These range from performing the same from copy-and-paste usage of standard methods regardless of the data's characteristics to applying an inappropriate statistical test to the analysis [4]. Furthermore, there is a tendency to publish only a limited statistical workflow and to focus exclusively on p-values and the software used, rather than on the exact methods used [5].

The innovative approach of the BioMedStatX software is to let the user perform reliable and reproducible statistics with critical statistical decisions being made by the application, to exclude most of the mentioned mistakes. Experimental data can be loaded into the application via Excel or CSV files. The software decides based on statistical assumption testing, if a parametric test can be performed or a non-parametric alternative need to be chosen, therefore it is not possible to mistakenly do a wrong test on a dataset. The user can choose from various tests offered by the program in case a transformation of the data or a post-hoc test is required. Only tests that are suitable for the respective test situation are suggested and the user is guided through this selection by the software. Because statistical significance alone does not imply biological relevance, BioMedStatX moreover reports effect sizes by default, making the magnitude of group differences transparent and helps users interpret their results more meaningfully [6,7]. Finally, the results are stored in a structured and detailed Excel workbook to ensure that all methodological information is stored in one accessible location.

Although multiple statistical software tools are available, such as GraphPad Prism (GraphPad Software, Boston, USA), JMP (JMP Statistical Discovery LLC, North Carolina, USA), and SPSS (IBM Corporation, New York, USA), these programs may require statistical expertise, depend on user choices without comprehensive guidance, or offer limited transparency in test selection. Consequently, statistical errors can still occur even when established software is used. BioMedStatX offers a combination of statistical accuracy and user-oriented design. Its features include automated test selection, assumption checks, and a transparent decision tree to help users follow valid statistical processes regardless of their background in statistics. Unlike many commercial tools, BioMedStatX is fully open-source, freely accessible, and designed to promote reproducibility and good scientific practice. An additional feature is its structured Excel export, which summarizes assumptions, results, test decisions, and effect sizes in a clear format suitable for both internal review and inclusion in scientific publications.

## Method details

BioMedStatX is used for statistical analysis and visualization of data, as typically found in biomedical and experimental studies. Its design reflects the statistical patterns and workflows that are characteristic for laboratory-based biomedical research. These include group-based comparisons, assumption-driven selection between parametric and non-parametric tests, repeated-measures designs, and transformation-guided decision-making. BioMedStatX therefore integrates the statistical procedures that are most commonly required for experiments in this field. These include, but are not limited to, RT-qPCR, western blot, ELISA, other biochemical assays such as viability, cytotoxicity, or apoptosis assays, flow cytometry as well as experiments with repeated measurements on the same specimen (morphology or behavioral analyses). It enables researchers to perform various statistical tests, interpret results, and create high-quality graphics for publications. It is Python-based, and both the program and the code are open access. The data to be analyzed should be available as an Excel or CSV file with clearly named columns. A template Excel file is provided with the program, to get an idea how the data should be structured.

The program automatically decides whether parametric or nonparametric tests have to be used, based on normality and homoscedasticity tests. Moreover, the application automatically applies the correct test depending on how many groups need to be compared (two or more groups). In contrast, widely used statistical environments such as SPSS, JMP, or GraphPad Prism offer large sets of analysis options that require substantial statistical or software-specific knowledge. SPSS often relies on syntax-based scripting for reproducible workflows or advanced analyses, which can be challenging for users without programming experience. JMP presents complex, multi-layered menus and extensive modeling options that may overwhelm inexperienced users when selecting a valid test. Even Prism, although more intuitive, allows users to manually choose any statistical test, including inappropriate ones, without enforcing prerequisite checks. BioMedStatX reduces this complexity by guiding users through a strictly validated workflow, enforcing assumption checks, restricting invalid test choices, and presenting only statistically appropriate options. This ensures that analyses remain simple, transparent, and reproducible, even for users with limited statistical training. The following tests are available up to now (Table 1):

**Table 1:** The following tests are included in BioMedStatX.

	Parametric	Non-Parametric
2 groups (independent)	*t*-test	Mann-Whitney-U test
2 groups (dependent)	paired *t*-test	Wilcoxon-Signed-Rank test
>2 groups (independent)	One-Way ANOVA	Kruskal-Wallis test
>2 groups (dependent)	Repeated-Measures ANOVA	-

Further tests:

➢ Transformations (log10, Box-Cox (*λ* estimated by Maximum-likelihood method), Arcsine-Square-Root)
➢ Welch’s *t*-test and ANOVA
➢ Mixed ANOVA and Two-Way ANOVA,
➢ Post-hoc tests (Tukey, Dunnett, Dunn’s, Šídák method)

**Fig. 1:**
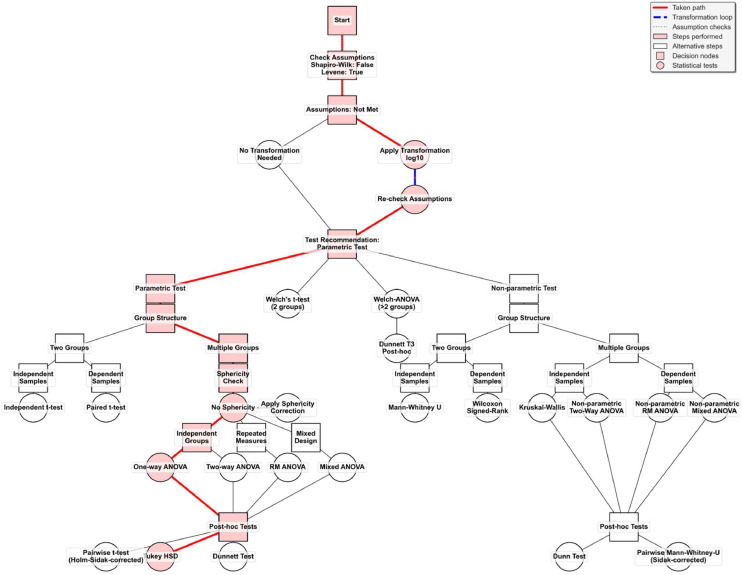
Decision tree in BioMedStatX. This decision tree helps the user to understand the statistical decisions made by the application and user. The tree adapts to the respective analysis and always highlights the tests that were performed.

Additionally, descriptive statistics on the data including mean, median, standard deviation (SD), standard error of the mean (SEM), confidence intervals, etc. for each group are provided. Cohen's d-effect size is calculated by default for all parametric comparisons using the pooled standard deviation for independent groups and the SD difference for paired measurements. The program can identify outliers in the data by either Grubbs' or the Modified-Z-score test and highlighting them accordingly. The user interface is based on PyQt5 and provides dialogs for configuring analyses and plots. Users can select groups, change the order, and configure the appearance and aesthetics of the plots. The program supports various plot types, including bar charts, box plots, violin plots, and raincloud plots. Colors, hatches, axis labels, titles, error bars, and other design elements can be customized. Statistical significance and post-hoc results are visualized directly in the plots using letters or bars. Plots can be saved in various formats (PNG, SVG). Tooltips and info dialogs guide the users through the application. Statistical results are exported as Excel workbooks with multiple sheets (Summary, Assumptions, Statistical Results, Descriptive Statistics, Decision Tree, Raw Data, Pairwise Comparisons, Analysis Log) and the statistical decisions are visually represented by a decision tree (comp. Fig. 1) that shows the statistical path that the user has taken together with the software. In the exported Excel file, the column “Group” contains the categorical experimental condition (e.g., treatment groups), whereas the column “Values” includes the numerical measurements associated with each data point. This distinction ensures that users can clearly interpret the structure of their processed dataset. Analysis logs and debug information are stored to ensure the traceability of the analyses. Additional functions can easily be added by the user as the most important components (statistics, visualization and GUI) are modular and can be expanded, e.g. with further statistical or post-hoc tests.

A typical workflow in BioMedStatX could look like this (A more detailed How-To including screenshots can be found in the GitHub repository):

1. Format the experimental data in a long format in an Excel or CSV file.
2. Load the data into the application via the “Browse…” button. Then choose the correct excel sheet and the group and value column. Available groups are synchronized automatically in the program.
3. The user must choose the groups to be analyzed. It is possible to create several analyses including different groups with the loaded data. Before starting the analysis, the plots can be customized if needed. It is also possible to perform the analysis without creating plots and just receiving the Excel file.
4. Analysis is started either via the “Create and analyze all plots” or “Create selected plot” button
5. After starting the analysis, the application first checks the number of groups that should be compared. Next, the normal distribution and homoscedasticity of the data are validated by Shapiro-Wilk-test and Levene-test. With these results, the program decides if a parametric test is possible or if the data needs to be transformed.
6. If a transformation is needed, the user can choose between Log10, Box-Cox (*λ* estimated by Maximum-Likelihood method) and Arcsine Square Root transformation.
7. Following the transformation, normal distribution and homoscedasticity are checked for the transformed data. Now the application decides for a parametric or non-parametric test. In this case, the application decides for a parametric test (ANOVA).
8. After performing a significant ANOVA, the user can choose between the Tukey-HSD, Dunnett and Šídák-method, where the user can select pairs of means, as post-hoc tests.

The user only intervenes twice in the analysis, when selecting the transformation and when selecting the post hoc test. The statistical results are automatically exported to Excel.

## Method validation

To verify the accuracy of the software, we analyzed an example dataset in BioMedStatX v1.0.0, JMP® v10.0.0 and GraphPad Prism 10 v10.5.0 and compared the results. Results are shown in Table 2. The example dataset can be found in the supplementary data (Supplements data S1). The results indicate that the analyses performed by all applied programs are highly consistent. Although minor differences may occur in presenting the significance level (p-value) of the One-Way ANOVA, all programs revealed identical F-statistic values.

**Table 2:** Comparison of test results between BioMedStatX, JMP® and GraphPad Prism 10. The same dataset was analyzed in BioMedStatX, JMP® and GraphPad Prism 10 using the same test execution order. Wild-type (WT), Knock-Out (KO), Knock-In (KI).

Method	BioMedStatX (v1.0.0)	JMP® (v10.0.0)	GraphPad Prism 10 (v10.5.0)
Shapiro-Wilk test	*p* = 0.0534	*p* = 0.0534	*p* = 0.0534
Brown-Forsythe test	*p* = 0.8746	*p* = 0.8746	*p* = 0.8746
One-Way ANOVA	*p* < 0.001 F-statistics: 217.91	*p* < 0.0001 F-statistics: 217.91	*p* < 0.0001 F-statistics: 217.9
Tukey-HSD	KI vs. KO *p* < 0.001 KI vs. WT *p* < 0.001 KO vs. WT *p* < 0.001	KI vs. KO *p* < 0.001 KI vs. WT *p* < 0.001 KO vs. WT *p* < 0.001	KI vs. KO *p* < 0.0001 KI vs. WT *p* < 0.0001 KO vs. WT *p* < 0.0001

With a second example dataset from a cell viability assay, we wanted to emphasize the necessity of applying the correct statistics on data. We analyzed a dataset (Supplements data S2) that did not meet the assumptions for a parametric test. Each group was tested individually with the Shapiro-Wilk test to determine whether the initial data in any group deviated from normal distribution in order to decide whether a transformation was necessary at all. After the values had been transformed, a normality check was applied to the model residuals because the residuals summarize all groups, after subtracting the group effect, and must be normally distributed for the ANOVA model assumptions to be valid. Despite transformation, the prerequisites did not allow for a parametric test (Table 3).

**Table 3:** Results of the Shapiro-Wilk test and Brown-Forsythe test. Tests were performed before a transformation, after log10 transformation and after Box-Cox transformation in BioMedStatX.

Method	Without transformation	After log10 transformation	After Box-Cox transformation
Shapiro-Wilk test	***ctrl****p* = 0.4803 ***A****p* = 0.0553 ***B****p* = 0.0048 ***C****p* = 0.3310 ***D****p* = 0.0147	*p* = 0.0003	*p<* 0.0001
Brown-Forsythe test	*p* = 0.0121	*p* = 0.8746	*p<* 0.0001

**Fig. 2:**
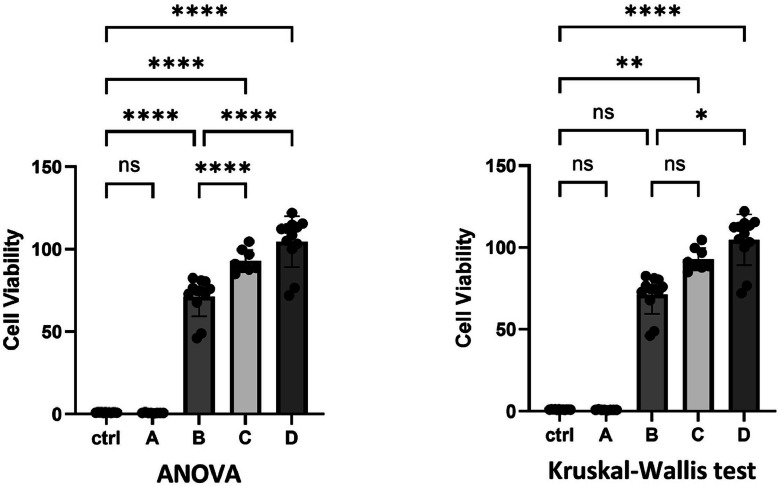
Different significances when testing with parametric and non-parametric tests. The graphs were created using GraphPad Prism 10, as BioMedStatX cannot create graphs or perform statistical analyses if the test does not meet the assumptions of the data. Data = mean ± SD; n = 8–12; * p < 0.05, ** p < 0.01, **** p < 0.0001 as indicated.

However, GraphPad and JMP still offer the option of using a parametric test on this data set. It is evident (Fig. 2), that the significance levels do not only vary between the two tests but there are also significant comparisons found in the case of parametric testing that are absent when applying the correct statistics for these data such as the Kruskal-Wallis test. If an incorrect test is carried out, this can result in the continuation of work based on erroneous assumptions, as the incorrect significance can be misleading. The Šídák method following the ANOVA shows significances (ctrl vs. B, B vs. C) that are not present when performing the Kruskal-Wallis test followed by the Šídák method. This underscores the significance of selecting the appropriate test based on the characteristics of the data. In BioMedStatX, the application automatically decides for a transformation, and the user only needs to choose one of the three types of transformations. If the transformation is successful, a parametric test is performed after transformation. Otherwise, a non-parametric test is carried automatically by the application. This is just one example of the decision-making process that is taken over by the application.

### Limitations

The same statistical parameter, e.g. normal distribution can be obtained with different tests. The normal distribution, for example, can be tested by the Bartlett- [8], Levene- [9], O’Brien- [10] or Brown-Forsythe test. We did not implement every possible test, but only the Brown-Forsythe test because it is more robust against outliers and non-normally distributed data, as the median it utilizes is less susceptible to extreme values [11]. Due to the open-source structure, it is of course possible to add more tests to the application at any time. Furthermore, non-parametric alternatives for Two-Way-, Mixed-Model- and Repeated-Measures-ANOVAs cannot be performed in BioMedStatX today. There are alternative tests available, but studies have made reservations about the use of certain tests, including the ART-ANOVA [12] and Friedmann test [13]. These tests have been found to frequently result in Type-I errors and have been demonstrated to only perform reliably under very specific conditions. New versions of BioMedStatX will be uploaded to the repository. Updates may include additional test options and enhancements to application usability. The BioMedStatX code is open source and can be modified by users who have specific requirements for tests or statistical methods.

## Ethics statements

None.

## CRediT author statement

**Philipp Krumm**: Conceptualization, Writing-Original draft preparation, Methodology, Software

**Nicole Böttcher**: Supervision, Conceptualization, Validation, Writing-Reviewing and Editing

**Richard Ottermanns:** Conceptualization, Writing-Reviewing and Editing

**Thomas Pufe**: Resources, Funding acquisition

**Athanassios Fragoulis**: Supervision, Conceptualization, Validation, Writing-Reviewing and Editing

## Related research article

None.

## Declaration of generative AI and AI-assisted technologies in the writing process

During the preparation of this work the authors used Chat GPT 4.0 in order to improve readability and language. After using this tool/service, the authors reviewed and edited the content as needed and take full responsibility for the content of the publication.

**Key innovations**

Intelligent test selection prevents incorrect statistical choices.

Automated assumption checks and guided data transformations.

Interactive graphical user interface (GUI) with comprehensive Excel export.

Alt-text: Unlabelled box

## Declaration of competing interest

The authors declare that they have no known competing financial interests or personal relationships that could have appeared to influence the work reported in this paper.

## Data Availability

I have shared the link to my data/code at the Attach File step.

## Appendix Supplementary materials

Image, application 1

Supplementary material *and* additional information Image, application 2
